# Target product profiles for pan-Africa recombinant antivenoms against neurotoxic or hemotoxic and cytotoxic snakebite envenoming

**DOI:** 10.1371/journal.pntd.0012833

**Published:** 2025-01-24

**Authors:** Andreas H. Laustsen, Melisa Benard-Valle, Abdulrazaq G. Habib, Nicholas R. Casewell, Michael Abouyannis, David G. Lalloo, Anne Ljungars

**Affiliations:** 1 Department of Biotechnology and Biomedicine, Technical University of Denmark, Kongens Lyngby, Denmark; 2 College of Health Sciences, Bayero University, Kano, Nigeria; 3 Centre for Snakebite Research and Interventions, Department of Tropical Disease Biology, Liverpool School of Tropical Medicine, Pembroke, Liverpool, United Kingdom

Snakebite envenoming is a neglected tropical disease that yearly causes more than 100,000 deaths worldwide and leaves many more individuals permanently disabled [1]. Because of this enormous human suffering, the World Health Organization (WHO) listed snakebite envenoming as a high-priority neglected tropical disease in 2017 [2], with the goal to reduce mortality and disability caused by snakebite envenoming by 50% by 2030 [3]. One of the avenues to reach this goal involves improving current envenoming therapy and developing new types of antivenom products. Today, the only specific treatments for snakebite envenoming are plasma-derived immunoglobulins or fragments of these (traditional antivenoms), which are obtained from large animals, such as horses, hyperimmunized with whole venoms from one or more snake species [4]. Although these traditional antivenoms continue to save lives, they come with some drawbacks, including limited efficacy against certain venoms or toxins, dependence on venoms for production, high production cost, batch-to-batch variation, and the risk of causing adverse immunological reactions upon administration [5]. To overcome these drawbacks, the development of recombinant antivenoms is being investigated [6]. These new types of antivenom are composed of broadly neutralizing, recombinantly produced antibodies or antibody fragments, and, today, several examples of how these can be discovered and optimized have been reported [7–11]. It is expected that, when properly engineered and developed, recombinant antivenoms have the potential to be safe, effective, and affordable [12,13]. However, to prove this in a clinical setting, the next step would be to develop and formulate specific antivenom products. To this end, decisions must be taken on desired product characteristics and properties, which can be outlined in so-called target product profiles (TPPs) (Fig 1). Such TPPs may also facilitate a regulatory harmonization of recombinant snakebite therapies in a centralized approach through the African Medicines Agency (similar to the European Medicines Agency) and thereby enable/expedite a timely introduction of products throughout sub-Saharan Africa [14].

**Fig 1:**
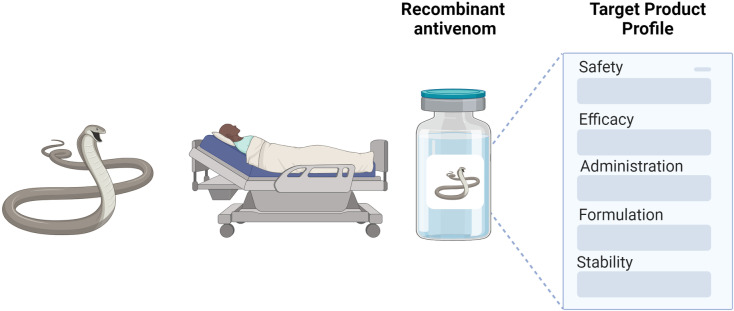
The development of recombinant antivenom products can be guided by understanding the desired characteristics and properties, which are typically outlined in so-called target product profiles (TPPs). The figure was created with BioRender.

As part of the WHO’s work within snakebite envenoming, recommended TPPs for traditional plasma-derived antivenoms for sub-Saharan Africa were developed and published in 2023 [15]. In these TPPs, key characteristics were defined by a technical and scientific advisory group, supported by the WHO and the Drugs for Neglected Diseases initiative. Thereafter, the public was consulted to allow relevant stakeholders to contribute to the final drafts. However, for completely new types of envenoming therapies, such as recombinant antivenoms, no recommended TPPs are available, and with the field of recombinant antivenom research moving at an increasing speed, a dire need for such TPPs is now presenting itself. In light of this, we developed our own suggested TPPs for recombinant antivenoms for the region of sub-Saharan Africa to serve as an exemplary starting point for the general development of recombinant antivenom products (S1 Text, TPPs). In these TPPs, we outline different product requirements depending on whether the product is intended for the treatment of neurotoxic or hemotoxic/cytotoxic snakebite envenoming. To facilitate easy translation and adaptation for researchers and product developers, we based our TPPs on WHO’s TPPs for plasma-derived antivenoms [15,16], but adapted the requirements to reflect the advantages that are anticipated for recombinant antivenoms compared to plasma-derived products in regards to safety, dosing, and manufacturing. This adaptation was based on literature and advice from experts in the field, who are mentioned in the acknowledgments, to ensure inclusivity and practical feasibility. We hope our TPPs can serve as an inspiration in the field of antivenom development and function as a starting point for the development of specific antivenom products for sub-Sahara Africa that will hopefully reach snakebite victims in the future. We also hope that our work serves as a foundation for the development of TPPs for recombinant antivenoms in other regions, allowing new TPPs to be tailored by incorporating clinical expertise and insights into regional variations in snake venom.

## Supporting information

S1 Text Target product profiles for pan-Africa recombinant antivenoms against neurotoxic or hemotoxic and cytotoxic snakebite envenoming. (DOCX)
