# Supplementary material for: Target product profiles for pan-Africa recombinant antivenoms against neurotoxic or hemotoxic and cytotoxic snakebite envenoming
Source: PLoS Negl Trop Dis. 2025 Jan 24;19(1):e0012833. doi: 10.1371/journal.pntd.0012833 (PMC11759373; doi:10.1371/journal.pntd.0012833)
Supplement: S1 Text — (DOCX) [file pntd.0012833.s001.docx]

# **Supporting information**

# **Target Product Profiles for pan-Africa recombinant antivenoms against neurotoxic or hemotoxic and cytotoxic snakebite envenoming**

Andreas H. Laustsen^1*^, Melisa Benard-Valle^1^, Abdulrazaq G. Habib^2^, Nicholas R. Casewell^3^, Michael Abouyannis^3^, David G. Lalloo^3^, Anne Ljungars^1*^

^1^Department of Biotechnology and Biomedicine, Technical University of Denmark, Kongens Lyngby, Denmark

^2^ College of Health Sciences, Bayero University, Kano, Nigeria

^3^Centre for Snakebite Research and Interventions, Department of Tropical Disease Biology, Liverpool School of Tropical Medicine, Pembroke Place, Liverpool, United Kingdom

^*^[aellj@dtu.dk](mailto:aellj@dtu.dk), [ahola@bio.dtu.dk](mailto:ahola@bio.dtu.dk)

# **Target Product Profile for pan-Africa polyvalent recombinant antivenoms for neurotoxic or hemotoxic/cytotoxic snakebite envenoming**

# Introduction

Snakebite envenoming is yearly causing more than 100,000 deaths worldwide and leaving many more maimed for life^1^. Currently, heterologous, animal plasma-derived immunoglobulins (aka traditional antivenoms) are used for treatment. These antivenoms are typically derived through hyperimmunization of large animals such as horses with whole venoms from one or more snake species. However, venoms are complex mixtures of proteins that vary in abundance and composition between and within snake species, and the proteinacious toxins vary in size, toxicity, and immunogenicity. Therefore, it is difficult to raise antibodies against some types of toxins through the immunization process used for the generation of traditional antivenoms, resulting in limited efficacy of the antivenom against some venoms/toxins. Furthermore, the production method for traditional antivenoms results in an Active Pharmaceutical Ingredient (API) comprising a mix of neutralizing and non-neutralizing antibodies, which limits the proportion of neutralizing antibodies in the final product compared to what can be achieved with recombinant methods. Other limitations of traditional antivenoms are their excessive cost for the target population, batch-to-batch variations, and the risk of causing adverse reactions upon administration due to their heterologous nature. To circumvent, or reduce, these limitations, there is an increased interest in recombinant antivenoms, consisting of recombinantly produced antibodies or antibody fragments. These recombinant antivenoms have the potential to be both safe, effective, and affordable^2,3^.

In addition, various strategies including phage display technology can be applied for the discovery of broadly neutralizing antibodies that neutralize multiple similar toxins or a whole toxin class^4,5^. By combining multiple such broadly neutralizing antibodies into oligoclonal mixtures^6^, broadly neutralizing antivenoms can be envisioned targeting single or multiple whole genera of snake species^7^. Based on the venom composition, which results in various clinical manifestations after envenoming, different antivenoms are needed. In the region of sub-Saharan Africa, WHO considers 24 species from 4 genera (*Bitis*, *Dendroaspis*, *Echis,* and *Naja*) to be of highest (category 1) medical importance^8^. The clinical syndromes of envenoming in sub-Saharan Africa are well-defined and the syndromic grouping of species is potentially useful in the diagnosis and management of snakebite envenomings. WHO guidelines define six clinical syndromes^9^, which, combined with necrosis, can be can be summarized as^10^:

1. Marked local swelling with coagulable blood: typically caused by bites from cytotoxic spitting cobras (*Naja* spp.), puff adders (*Bitis arietans*), and (southern Africa only) Berg adders (*Bitis atropos*);
2. Marked local swelling and necrosis: typically caused by bites from cytotoxic spitting cobras (*Naja* spp.);
3. Marked local swelling with incoagulable blood and/or spontaneous systemic bleeding: most typically caused by bites from carpet vipers (*Echi*s spp.) in sub-Saharan Africa or, in the Sahara Desert, by desert horned vipers (*Cerastes cerastes*). More uncommonly it may sometimes follow bites by bush vipers (*Atheris* spp.), puff adders (*B. arietans*), or gaboon vipers (*B. gabonica* and *B.rhinoceros*);
4. Progressive paralysis (neurotoxicity): due to bites by neurotoxic, typically non-spitting cobras (*Naja* spp.) and by mambas (*Dendroaspis* spp.);
5. Mild swelling alone: associated generally with bites by burrowing asps (*Atractaspis* spp.), night adders (*Causus* spp.), and by some species of dwarf, bush and desert vipers (*Atheris* spp., and *Cerastes* spp.).

This TPP focuses on a recombinant antivenom used for the treatment of patients with the clinical manifestations described in either:

- group 1, 2, 3 above which is polyvalent and neutralizes non-neurotoxic (i.e., cytotoxic/hemotoxic) envenoming
- group 4 above which is polyvalent and neutralizes neurotoxicity caused by both non-spitting cobras (*Naja* spp.) and mambas (*Dendroaspis* spp.).

The TPP is adapted from the TPPs recommended by WHO for traditional antivenoms^10^. It is important to note that we envisage recombinant antivenoms to be comprised of a few broadly neutralizing human antibodies, fragments thereof and/or nanobodies that, thanks to their typically good safety profile, should rarely result in clinically significant adverse reactions.

# Scope

## Indication

**Neurotoxic envenoming**

For the treatment of snakebite envenoming by an identified or unidentified species of WHO Category 1 or 2 sub-Saharan African snake namely a neurotoxic species of cobra (*Naja* spp.) or mamba (*Dendroaspis* spp.) that results in a clinical syndrome of envenoming dominated by neurotoxic effects including for example, palpebral ptosis, bulbar muscle weakness and neuromuscular paralysis^1^, to be used in conjunction with supportive care, such as mechanical ventilation.

**Hemotoxic/cytotoxic envenoming**

For the treatment of snakebite envenoming by an identified or unidentified species of WHO Category 1 or 2 sub-Saharan African snake that results in a clinical syndrome of envenoming dominated by haemorrhagic, cytotoxic, or pro/anticoagulant effects (namely cytotoxic species of cobra (*Naja* spp.), African adder/viper (*Bitis* spp.), or carpet viper (*Echis* spp.), to be used in conjunction with other treatments to address disease manifestations.

## Contra indication

**Neurotoxic envenoming**

Hypersensitivity to the active substance or any of the excipients.

**Hemotoxic/cytotoxic envenoming**

Hypersensitivity to the active substance or any of the excipients.

## Target population

**Neurotoxic envenoming**

All envenomed patients with neurotoxic clinical manifestations in the region.

**Hemotoxic/cytotoxic envenoming**

All envenomed patients with coagulopathy, haemorrhagic effects, tissue necrosis, or other cytotoxic clinical manifestations in the region.

The antivenoms are used to treat snakebite envenoming in men, women (including pregnant women), and children of all ages.

## Geographic working range

Sub-Saharan Africa

## Intended end users

Health care professionals

## Implementation in the healthcare system

| Characteristics | Optimal | Minimal |
| --- | --- | --- |
| Level of implementation in the healthcare system | The product is capable of being  deployed at all levels of the health  system and can be deployed at independent rural sites by health staff trained in the diagnosis and treatment of snakebite envenoming. | The product is deployed to all  levels of the health system that  meet optimum criteria for  infrastructure and can be  administered by trained health  workers under the direct  supervision of an appropriately  qualified medical doctor. |
| *Comments:*  Antivenom is a time-critical emergency biotherapeutic product and should ideally be available as close to the communities in which people are at risk of snakebite envenoming as it is possible. Products defined by this TPP should have safety profiles that make them amenable to being deployed to primary health care facilities that have health workers who have been trained in the diagnosis and emergency treatment of snakebite envenoming. While it is preferable that antivenom will be administered under the direct supervision of an appropriately qualified  and experienced medical doctor, the use of antivenom under indirect (e.g.: following telephone consultation, radio communication, or other “telemedicine” engagement with the medical doctor) supervision should be encouraged as the optimal case for expanding rapid accessibility to safe, effective antivenoms for the majority of the population. Minimal clinical skills for health workers administering antivenoms should include: ability to detect criteria for antivenom treatment (clinical signs of neurotoxic and hemotoxic/cytotoxic envenoming), perform and interpret bedside tests, gain intravenous access, and detect signs of early adverse reactions caused by the administered antivenom and treat with adrenaline/epinephrine and antihistamines as appropriate. | | |

# Manufacturing considerations

## Active pharmaceutical ingredient

Whole immunoglobulins (IgGs), fragments antigen-binding (Fabs), single domain antibodies, nanobodies (V_H_Hs), or fusion proteins such as multiple V_H_Hs linked together or V_H_H fused to a human Fc-domain, and/or combinations of these.

## Specific active pharmaceutical ingredient

| Characteristics | Optimal | Minimal |
| --- | --- | --- |
| Specific active pharmaceutical ingredient | Not less than 98% of the total  protein content must consist of intact active pharmaceutical ingredient. i.e., neutralizing antibodies or antibody fragments that are specific against snake toxins | Not less than 92% of the  total protein content must consist  of intact active pharmaceutical  ingredient. i.e., neutralizing antibodies or antibody fragments that are specific against snake toxins |
| *Comments:*  Specific active pharmaceutical content refers to the amount of the defined active pharmaceutical ingredient (API) for the product, i.e., the recombinant IgG, Fab, V_H_H, V_H_H constructs, or combinations thereof.  The purity of antivenom is linked to product safety, tolerability, and efficacy, and recombinant antivenoms are expected to consist of a high degree of specific pharmaceutical ingredient of high purity.  It is to be noted that for recombinant antivenoms, all antibodies or antibody fragments will be toxin-targeting and neutralizing, which may not be the case for plasma-derived antivenoms, where only a fraction of the antibodies or antibody fragments target snake toxins. However, even for recombinant antivenoms, a small fraction of the antibodies might be denatured, aggregated, or degraded upon formulation and storage, resulting in less than 100% of the protein content being API. The API can be determined using for example a combination of concentration determination, size-exclusion chromatography, ELISA, and/or mass spectrometry. | | |

## Finished product forms

A lyophilized final product form is preferable to achieve the stability requirements. Standard excipients for antibody/nanobody-based product formulation should be chosen to ensure high stability and sterility. Stability of the product for the desired duration (whether lyophilized or liquid) should be validated by appropriate stability testing, preferably using both real time and accelerated stability studies.

*Comments*

Both lyophilized and liquid preparations have advantages and limitations.

Current liquid preparations dispensed in the final container under GMP compliant conditions are easier to use clinically but require the guarantee of storage and transportation under conditions maintaining a cold chain (typically 2-8 °C).

Lyophilized formulations may usually be transported and stored at a temperature not exceeding 25 °C and are of interest for distribution to areas where the cold chain cannot be guaranteed, such as in many tropical regions of the world. However, lyophilization/fill/finish is an expensive and complex manufacturing operation that should be carefully validated and operated to maintain the quality of the product. Faulty lyophilization can result in denatured protein that is difficult to solubilize.

Many of the countries where these products are deployed are considered to be ICH climatic zone III, IVa, or IVb. Both liquid and lyophilized forms should ideally tolerate a temperature of at least 30 °C (ideally 40 °C) and the seal of the vials should be resistant to a relative humidity up to 90%.

## Price

Principles of “fair pricing” should guide discussion between buyers and sellers, sometimes referred to as the lowest possible sustainable price. Recombinant antivenoms are expected to be produced at the same or at lower costs for a treatment course compared to traditional plasma-derived antivenoms^3^.

# Performance

## Preclinical efficacy

For assessing preclinical efficacy, a standard or venom pool should be used for challenge, prepared from specimens of the venomous species expected to cause accidents in the region intended for its use.

| Characteristics | Optimal | Minimal |
| --- | --- | --- |
| Preclinical efficacy | Preclinical potency and toxin-specific  activity bioassays with whole venoms demonstrate the potential of the antivenom to neutralize, *in vivo,* 2 times the average adult venom yield of the whole venoms from all the neurotoxic or non-neurotoxic (hemotoxic/cytotoxic), medically relevant species in a specified region species.  Prevent lethality *in vivo* in preincubation mice models including all the whole venoms the product is neutralizing. This assay is to be performed both during the pre-clinical development and as a quality control assay of the final product.  Minimal batch-to-batch variation where every batch has a defined composition and predictable ratio of potency against all species that it is described to neutralize.  **Neurotoxic:**  During pre-clinical development, in addition to preincubation assays, also show that the antivenom can prevent lethality *in vivo* in rescue mice models^11,12^ where first 2-3 LD_50_s of the whole venom is given *s.c*. or *i.m.,* followed by immediate *i.v.* injection of the recombinant antivenom. This should be conducted pre-clinically with all the neurotoxic whole venoms the product is neutralizing.  **Hemotoxic/cytotoxic:**  During pre-clinical development, in addition to lethality also show, when relevant, that the antivenom can reduce procoagulant and/or anticoagulant activity, hemorrhagic activity, and dermonecrotic activity *in vivo* in preincubation mice models  including all the whole venoms from all the hemotoxic/cytotoxic medically relevant species in a specified region that the product is neutralizing. These assays are to be performed both during the pre-clinical development and as a quality control assay of the final product.  In addition to preincubation assays, also show, when relevant, that the antivenom can reduce procoagulant and/or anticoagulant activity, hemorrhagic activity, and dermonecrotic activity *in vivo* in rescue mice models where the whole venom is given *s.c*. or *i.m.,* followed by immediate *i.v*, or possibly *s.c*. or *i.m.* injection of the recombinant antivenom,  including all the whole venoms from all the hemotoxic/cytotoxic medically relevant species in a specified region that the product is neutralizing. | Preclinical potency bioassays  demonstrate the potential of the  antivenom to neutralize, *in vivo,* the average adult venom yield of the whole venoms of all neurotoxic or non-neurotoxic (hemotoxic/cytotoxic) species for which efficacy is claimed.  Prevent lethality *in vivo* in preincubation mice models including all the whole venoms the product is neutralizing. This assay is to be performed both during the pre-clinical development and as a quality control assay of the final product.  Minimal batch-to-batch variation where every batch has a defined composition and predictable ratio of potency against all species that it is described to neutralize. |
| *Comments:*  To evaluate the lethal effect, the potency [P] is derived from ED_50_ and LD_50_ and is the amount of venom neutralized (in mg) per mg of antivenom [mg V neutralized/mg antivenom]) using the equation^13^:  $P=\frac{(n-1){LD}_{50}}{{ED}_{50}}$  Where “n” is the number of LD_50_ used in the determination of the ED_50_, *P* is the amount of venom (V) that is completely neutralized per mg unit of antivenom, and would protect 100% of mice, as opposed to ED_50_ which is the amount that protects 50% of them. The expression “(n-1) LD_50_” is used instead of the total amount of venom (n LD_50_) because at the endpoint of the neutralization assay, one LD_50_ (n=1) remains unneutralized and causes the death of 50% of mice. To transform the neutralization activity from ED_50_ to *P*, LD_50_ should be expressed as [mg venom/mouse] and ED_50_ in [mg antivenom/mouse]. Potency enables estimation of the amount of antivenom required to provide complete neutralization of a given quantity of venom. This is more relevant than the ED_50_, since it estimates the dose for complete neutralization of lethality (and protection of all the test animals) rather than just protection of 50% of the test animals. Note that the neutralization capacity per mg will be dependent on the size (molecular weight) of the included antibody or antibody fragment. | | |

## Clinical efficacy

| Characteristics | Optimal | Minimal |
| --- | --- | --- |
| Clinical effectiveness  (Including selected outcome measures) | Clinical effectiveness supported by adequately controlled prospective clinical trials.  The likelihood of a clinical response is dependent on the time interval between the snakebite and the administration of the product. For neurotoxic and cytotoxic envenoming, the below criteria apply when the product is administered within 4 hours of the snakebite. For hemotoxic envenoming, the below criteria apply if the product is administered within 8 hours of the snakebite.  **Neurotoxic:**   - case fatality rate (CFR) to <1%. - The development of a need for artificial ventilation is averted in 90% of patients. - Signs of neurotoxicity (e.g., fasciculations, paralysis of limbs, palpebral ptosis, paralysis of respiratory muscles) improve within 24-hours of antivenom administration.   **Hemotoxic/cytotoxic:**   - case fatality rate (CFR) to <1%. - amputations to <1%. - reduce spontaneous systemic bleeding (e.g. from the gums). - abnormal blood coagulability profile is restored in 3-9 hours. - persistence of coagulopathy at 24 hours post-antivenom to <3%; - need for debridement of dead tissue and/or skin grafting (excluding decompression or   deroofing of blisters) to <5%;  and,   - residual disability at 6 months post-bite (e.g., contracture, arthrodesis, weakness, inability to walk unaided after lower limb bites, or the requirement for renal or hormonal replacement therapy) to <5%. | For hemotoxic/cytotoxic envenoming, efficacy should be at least equivalent to a recommended and approved animal derived antivenom product. This efficacy should be demonstrated in a parallel group clinical trial.  For neurotoxicity, the feasibility of conducting a clinical trial in sub-Saharan Africa for any product is uncertain. To date, there has never been a clinical trial of neurotoxic envenoming in this region^14^. Therefore clinical effectiveness for a neurotoxic antivenom treatment can be supported by appropriate observational clinical data. There should be evidence that the product is at least equivalent in efficacy to a recommended and approved animal derived antivenom product. |
| *Comments:*  The clinical effectiveness of an antivenom can only be assessed under the following conditions:  1. It is used for treatment of envenoming by a snake species whose venom it is neutralizing (i.e., it is specific for that species)  2. It is given in an appropriate initial dose, that is optimally based on findings of clinical trials, or at a minimum, the results of independent pre-clinical testing by a competent laboratory and has been accepted and is recommended by national regulators, or in national/regional guidelines.  3. It is given within an acceptable time frame after the bite (i.e.: optimally within 4 hours or minimally within 8 hours).  Characteristics for minimal parameters were defined based on published reports of the performance of past and present traditional antivenoms such as FAVAfrique, EchiTab Plus ICP, IPSER-Africa, and SAIMR polyvalent antivenom^10^and EchiTab G monovalent antivenom.  Characteristics for optimal performance aim for an improvement over and above what is currently best-in-market performance of at least 50%, based on adoption of TPPs into manufacturing of new or improved products.  Importantly, the clinical effectiveness is affected also by the healthcare system itself, the access to trained medical personnel, etc. which are factors un-related to the antivenom itself.  To enable rapid antivenom administration, preferably within 4 hours post-bite, access to emergency healthcare is critical. | | |

## Safety and tolerability

| Characteristics | Optimal | Minimal |
| --- | --- | --- |
| Safety and tolerability | No or rare mild infusion related reactions (IRRs) grade 1 or 2, in <5% of the patients. Moderate or severe IRRs not present, or present in <1% of patients.  Standard toxicology evidence of safety in healthy adults.  Evidence of product safety in special populations including children, elderly patients, and pregnant women. | Mild infusion related reactions (IRR) in <20% of the patients. Moderate or severe IRRs grade 3 or 4 including bronchospasm and hypotension requiring treatment, cardiac dysfunction, anaphylaxis, and other symptoms observed in <2.5% of the patients.  Standard toxicology evidence of safety in healthy adults. |
| *Comments:*  Based on the production method used for recombinant antivenoms these are expected to be of high purity and display similar good safety profiles as therapeutic recombinant human or humanized antibodies, antibody fragments, or nanobodies used for other indications than snakebite envenoming. Based on adverse events reported for an antibody mixture used for rabies treatment^15^ and some therapeutic antibodies used in different fields, such as for cancer treatment targeting endogenous targets, some IRRs can occur^16,17^, although severe adverse effects are unlikely.  Although not recommended, due to the good safety profile of the product, it should be safe to apply even in the absence of snakebite envenoming, which might be beneficial to provide treatment before potentially irreversible complications of envenoming occur, or in cases where clinical signs of envenoming are unclear. | | |

| Characteristics | Optimal | Minimal |
| --- | --- | --- |
| Interaction with other medical products | There are no interactions with other medicinal products. | There are no serious interactions with other medicinal products,  and only minimal minor interactions. |

# Clinical pharmacology

## Clinical studies

Well-designed, pragmatic, and transparently managed clinical trials of antivenom are essential, and

the antivenoms should be carefully evaluated in both pre-clinical laboratory studies and in clinical trials prior to marketing authorization or licensing. Clinical trials need to adhere to the principles of Good Clinical Practice (GCP), an international ethical and scientific quality standard for designing, conducting, recording, and reporting trials that involve the participation of human subjects. Compliance with this standard provides public assurance that the rights, safety, and well-being of trial subjects are protected, consistent with the principles that have their origin in the Declaration of Helsinki, and that the clinical trial data are credible.

## Dosing and administration

All patients regardless of age, sex or body weight should receive the same dose. Using informed data of toxin antibody binding and inhibition data, the initial dose will be estimated based on preclinical pharmacology and toxicology studies and will be further refined in clinical studies^18^. Additional doses may be administered based on the observed clinical picture and considering the natural time course for the reversal of functional and physiological disturbances.

*Comments:* Venomous snakes do not meter the dose of injected venom according to the size or weight of bitten persons, and it is currently not possible to quantitatively measure the concentration of injected venom in patients at the bedside to inform dosing decisions. Hence all patients need to receive the same, standardized initial dose of antivenom, one which is adequate to neutralize all injected venom based on preclinical pharmacology and toxicology studies and refined in clinical studies^18^. The dose recommendations on package inserts should be based on adequately designed clinical trials, which consider the relatively low incidence of human cases in many regions.

| Characteristics | Optimal | Minimal |
| --- | --- | --- |
| Route of administration | Administered by intravenous infusion and have alternative administration routes of intramuscular or subcutaneous injection. | Administered by intravenous infusion. |
| *Comments:*  Effectiveness when using alternative routes of administration will depend on the size and format of the antibodies or antibody fragments present in the antivenom, and therefore their absorption. Moreover, effectiveness may also depend on the type of toxins in the venom injected and the site of injection. | | |

## Frequency of administration

| Characteristics | Optimal | Minimal |
| --- | --- | --- |
| Frequency of administration | Administration of a single dose of antivenom. This dose should be established through well-designed and administered randomized controlled trials.  The dose should neutralize lethality and hemotoxic/cytotoxic effects of each of the snake species for which the product is intended.  No recurrence of envenomation symptoms observed at any time after the initial dose. | Administration of a single dose of antivenom. This dose should be established through well-designed and administered randomized controlled trials.  Additional doses may be given based on the half-life in circulation of the product and the observed clinical picture over time. |
| *Comments:*  Snakebite is a time-critical emergency, and the sooner that a fully effective dose of an appropriate antivenom is administered, the better the chance that the patient will have a good outcome with minimal sequelae. This is best achieved by ensuring that every patient who has clinical signs and symptoms sufficient to warrant administration of antivenom receives a primary (initial) dose that is able to neutralize all of the injected venom. Since data is lacking on the masses of venom injected in real cases of snakebite envenoming, the most appropriate, available proxy (and one that is used elsewhere in the world) is average adult mass by weight of venom collected from specimens of each species during manual venom extraction. We suggest that WHO coordinates collection of data on average adult venom yields and include this data in its Guidelines for the Production, Control and Regulation of Snake Antivenom Immunoglobulins. | | |

## Product stability and storage

| Characteristics | Optimal | Minimal |
| --- | --- | --- |
| Product stability | For lyophilized products: At least 5 years in conditions up to  and including ICH climatic zone IVb  (ideally also temperatures of 40 °C and a relative humidity of up to 90%, which are reached in certain snakebite endemic settings).  The stability following reconstitution should ideally be 3 hours at conditions including ICH climatic zone IVb (temperature of 30 °C and relative humidity of up to 75%) or in refrigerator. | For lyophilized products: At least  3 years in conditions up to and  including ICH climatic zone IVb  (temperature of 30 °C and  relative humidity of up to 75%).  The stability following reconstitution should ideally be more than 2 hours in conditions including ICH climatic zone IVb (temperature of 30 °C and relative humidity of up to 75%.  For liquid products: At least 3  years in conditions when refrigerated (temperature of 2-8 °C). |
| *Comments:*  Countries in sub-Saharan Africa have climates that range from ICH climatic zone III to IVb. Products deployed in these regions should be able to tolerate the maximum threshold (IVb). Longer shelf lives are preferred. Both real-time and accelerated stability studies should be considered to establish the thermal tolerances of antivenoms, ideally, liquid products should be able to withstand an interruption of the cold chain of up to 24 hours and lyophilized products should be able to be stored at temperatures above 3-8 °C. | | |

| Characteristics | Optimal | Minimal |
| --- | --- | --- |
| Characteristic reconstitution time | Less than 5 minutes. | Less than 10 minutes. |
| *Comments:*  Only applicable for lyophilized products. The reconstitution time will be a function of the quality of lyophilization and the choice of excipients. | | |

| Characteristics | Optimal | Minimal |
| --- | --- | --- |
| Storage | For both lyophilized and liquid products: thermostability at ICH climatic zone IVb. Room temperatures at up to 30°C and relative humidity of up to 75%. | For lyophilized products: Room temperatures at up to 30 °C and relative humidity of up  to 75% or refrigerated cold chain storage at 2-8 °C.  For liquid products: refrigerated cold chain storage at 2-8 °C. |

| Characteristics | Optimal | Minimal |
| --- | --- | --- |
| Presentation | A single container (e.g.: vial,  ampoule, or intravenous infusion bag) that holds sufficient active  pharmaceutical ingredient to  neutralize envenoming from all the snake species for which it is intended. | A single container (e.g.: vial, ampoule, or intravenous infusion bag) or a carton/box that is clearly labelled as containing vials that must be given together in order to constitute a clinically effective initial dose comprising sufficient active pharmaceutical ingredient for the snake species for which it is intended. |

| *Comments:*  The presentation of antivenoms in vials or ampoules that do not contain a complete therapeutic dose contributes to the systematic under-dosing of patients in many settings, but especially in those where out-of-pocket spending remains the main source of funding for antivenom treatment. In order to ensure that all patients receive an effective therapeutic dose of antivenom as early as possible, and to minimize the possibility that economic pressure contributes to under-dosing, especially in situations where the cost is borne by the patient, presenting the antivenom as a single effective therapeutic dose (e.g.: in vials or sterile infusion bags) can achieve these objectives. The amount of total protein, the amount of API, and the specific amounts of any other vial contents (e.g.: aggregates, other proteins, etc.) should be included on vial labels and other packaging. |
| --- |

## Packaging

Each outer package (e.g.: box or carton) should contain one complete initial dose, presented in a single container (e.g.: vial, ampoule, or intravenous infusion bag). Lyophilized presentations should be accompanied by an adequate volume of isotonic fluid or sterile water for injection (WFI) to ensure complete reconstitution of the product. Package inserts should be provided in the language of the country where the product is being marketed. Inserts should meet the requirements of internationally accepted guidelines (e.g., ICH, WHO) and national regulations in the country of manufacture and the country where the product will be marketed. Information on the total protein content and the total active pharmaceutical ingredient (API) content should be included on vial labels and package inserts.

*Comments:*

In line with the characteristics for product presentation as a single vessel dose, outer packaging should clearly indicate single use/single dose. Solutions provided for reconstitution of lyophilized antivenoms must be produced in competent GMP environments, be sterile, appropriately packaged and correctly labelled.

# Operational characteristics

## Supportive adjunctive therapy

Essential ancillary drugs and consumables needed to treat snakebite envenoming include adrenaline/epinephrine, antihistamines, corticosteroids, antibiotics, paracetamol and other non-NSAID analgesics, iv fluids, iv giving sets, syringes, needles, iv cannulas, and related consumables. The monitoring of basic vital signs (heart rate, blood pressure, and respiratory rate) is essential in all cases. Pulse oximetry and equipment for respiratory support is desirable.

*Comments:*

The items listed should be available for potential use with all patients who present with a suspected snakebite. The items shown are an essential list for any setting in which antivenom is being administered. Depending on individual case presentation some patients will need to be managed in

facilities with substantially greater resources.

## Training & educational needs

Knowledge of common local snake species including non-venomous ones, history and clinical examination, criteria for antivenom treatment, monitoring vital signs (including orthostatic BP), POC tests (20WBCT, urine reagent sticks), resuscitation of shocked patients, nursing sick patients (positioning), iv access, iv cannula placement, management of iv infusion, criteria for use of/administration of adrenaline/epinephrine and other ancillary drugs.

*Comments:*

There is a need for improved clinical training of health workers in the diagnosis, treatment, and management of patients with real or suspected snakebite envenoming. Medical schools, nursing, and health worker training colleges should be encouraged and supported to incorporate more detailed teaching on snakebite envenoming into curricula, countries should work with professional bodies and subject matter experts to develop standardized national or regional guidelines.

# References

1. Gutiérrez, J. M. *et al.* Snakebite envenoming. *Nat Rev Dis Primers* **3**, 17063 (2017).

2. Thumtecho, S., Burlet, N. J., Ljungars, A. & Laustsen, A. H. Towards better antivenoms: navigating the road to new types of snakebite envenoming therapies. *J Venom Anim Toxins Incl Trop Dis* **29**, e20230057.

3. Jenkins, T. P. & Laustsen, A. H. Cost of Manufacturing for Recombinant Snakebite Antivenoms. *Frontiers in Bioengineering and Biotechnology* **8**, (2020).

4. Ledsgaard, L. *et al.* Discovery and optimization of a broadly-neutralizing human monoclonal antibody against long-chain α-neurotoxins from snakes. *Nat Commun* **14**, 682 (2023).

5. Ahmadi, S. *et al.* An *in vitro* methodology for discovering broadly-neutralizing monoclonal antibodies. *Sci Rep* **10**, 10765 (2020).

6. Laustsen, A. H. *et al.* *In vivo* neutralization of dendrotoxin-mediated neurotoxicity of black mamba venom by oligoclonal human IgG antibodies. *Nat Commun* **9**, 3928 (2018).

7. Sørensen, C. V. *et al.* Discovery of a human monoclonal antibody that cross-neutralizes venom phospholipase A_2_s from three different snake genera. *Toxicon* **234**, 107307 (2023).

8. Guidelines for the production, control and regulation of snake antivenom immunoglobulins, Annex 5, TRS No 1004. https://www.who.int/publications/m/item/snake-antivenom-immunoglobulins-annex-5-trs-no-1004.

9. World Health Organization. Regional Office for Africa. *Guidelines for the Prevention and Clinical Management of Snakebite in Africa*. (World Health Organization. Regional Office for Africa, 2010).

10. Target product profiles for animal plasma-derived antivenoms: antivenoms for treatment of snakebite envenoming in sub-Saharan Africa. https://www.who.int/publications-detail-redirect/9789240074569.

11. Knudsen, C. *et al.* Novel Snakebite Therapeutics Must Be Tested in Appropriate Rescue Models to Robustly Assess Their Preclinical Efficacy. *Toxins* **12**, 528 (2020).

12. Sørensen, C. V. *et al.* Antibody-dependent enhancement of toxicity of myotoxin II from *Bothrops asper*. *Nat Commun* **15**, 173 (2024).

13. Morais, V., Ifran, S., Berasain, P. & Massaldi, H. Antivenoms: potency or median effective dose, which to use? *J. Venom. Anim. Toxins incl. Trop. Dis* **16**, 191–193 (2010).

14. Abouyannis, M. *et al.* Clinical outcomes and outcome measurement tools reported in randomised controlled trials of treatment for snakebite envenoming: A systematic review. *PLOS Neglected Tropical Diseases* **15**, e0009589 (2021).

15. McClain, J. B., Chuang, A., Reid, C., Moore, S. M. & Tsao, E. Rabies virus neutralizing activity, pharmacokinetics, and safety of the monoclonal antibody mixture SYN023 in combination with rabies vaccination: Results of a phase 2, randomized, blinded, controlled trial. *Vaccine* **39**, 5822–5830 (2021).

16. Baldo, B. A. Immune- and Non-Immune-Mediated Adverse Effects of Monoclonal Antibody Therapy: A Survey of 110 Approved Antibodies. *Antibodies* **11**, 17 (2022).

17. Rombouts, M. D., Swart, E. L., Eertwegh, A. J. M. V. D. & Crul, M. Systematic Review on Infusion Reactions to and Infusion Rate of Monoclonal Antibodies Used in Cancer Treatment. *Anticancer Research* **40**, 1201–1218 (2020).

18. Muller, P. Y., Milton, M., Lloyd, P., Sims, J. & Brennan, F. R. The minimum anticipated biological effect level (MABEL) for selection of first human dose in clinical trials with monoclonal antibodies. *Current Opinion in Biotechnology* **20**, 722–729 (2009).
